# Impact of Environmental Microplastic Exposure on Caco-2 Cells: Unraveling Proliferation, Apoptosis, and Autophagy Activation

**DOI:** 10.3390/ijerph22060922

**Published:** 2025-06-11

**Authors:** Hana Najahi, Nicola Alessio, Massimo Venditti, Gea Oliveri Conti, Margherita Ferrante, Giovanni Di Bernardo, Umberto Galderisi, Sergio Minucci, Mohamed Banni

**Affiliations:** 1 Laboratory of Agrobiodiversity and Ecotoxicology LR21AGR02, Sousse University, Chott-Mariem, Sousse 4042, Tunisia; 2 Higher Institute of Biotechnology, Monastir University, Monastir 1002, Tunisia; 3 Department of Experimental Medicine, “Luigi Vanvitelli” Campania University, 81038 Napoli, Italy; 4 Environmental and Food Hygiene Laboratory (LIAA), Department of Medical, Surgical Sciences and Advanced Technologies G. F. Ingrassia, Catania University, Via Santa Sofia 87, 95123 Catania, Italy

**Keywords:** microplastics, Caco-2 cells, oxidative stress, apoptosis, autophagy

## Abstract

Microplastics (MPs) are pervasive environmental pollutants that have raised concerns due to their potential toxic impacts on human health. This study investigates the impact of polyethylene (PE) and polyethylene terephthalate (PET) microplastics on Caco-2 cells, a commonly used in vitro model for the intestinal barrier. Caco-2 cells were exposed to MPs of different sizes (1 µm and 2.6 µm) for 72 h. The results demonstrated a significant decrease in cell viability, accompanied by increased reactive oxygen species (ROS) production, suggesting oxidative-stress-induced cytotoxicity. Flow cytometry and Western blot analyses revealed that the MPs induced apoptosis, as evidenced by an increased Bax/Bcl-2 ratio and caspase-3 activation. Additionally, MPs triggered autophagy, indicated by elevated LC3-II levels and decreased p62 expression. The use of bafilomycin A1 further confirmed the enhancement of autophagic flux. These findings highlight the potential cytotoxic effects of MPs on intestinal epithelial cells, raising concerns about their impact on human health.

## 1. Introduction

Plastics are among the most prevalent pollutants in various ecosystems, including lakes, oceans, rivers, and soil. A major environmental concern associated with plastic waste is its ability to break down into tiny particles known as microplastics, which measure less than 5 mm, and nanoplastics (NPs), which are only a few hundred nanometers or smaller. This fragmentation and degradation occur due to natural influences such as ultraviolet (UV) exposure and mechanical forces, as well as biological processes driven by microorganisms [1]. In recent years, microplastics have been detected worldwide [2,3]. Their presence in the environment has raised significant concerns, particularly after studies reported their occurrence in the human diet [4,5,6]. Recent research indicates that plastic particles can enter the human food chain through the consumption of seafood and land-based food products [7]. Indeed, microplastics have been detected in various food and beverage products, including bottled water, tea bags, beer, salt, sugar, honey, seafood, vegetables, and fruits [8,9,10,11,12]. For instance, studies estimate that bottled water can contain anywhere from 0 to 10,000 plastic particles per liter [13,14]. In fact, drinking water is recognized as a significant route through which MPs can enter the human body, as these particles have been detected in tap water [15]. Moreover, it has been estimated that humans may ingest and inhale between 74,000 and 121,000 MP particles annually [16]. Plastic particles as small as 700 nm have recently been detected and quantified in human blood, highlighting the absorption and bioavailability of MPs through different exposure pathways [17]. Furthermore, plastic particles as small as 700 nm have recently been detected and quantified in human blood, highlighting the absorption and bioavailability of MPs through different exposure pathways [17]. The Caco-2 cell line, established from human colorectal adenocarcinoma [18], is widely used as an in vitro model for the human intestinal epithelium. Upon reaching confluence, these cells spontaneously differentiate, acquiring characteristics typical of absorptive enterocytes, such as tight junctions, brush-border enzymes, and active transport systems [19,20,21]. Their physiological relevance has made them a reference model for predicting human intestinal absorption and evaluating the impact of compounds on intestinal barrier function [22]. Despite this, the effects of microplastics on human health remain poorly understood. After ingestion, microplastics first come into contact with the intestinal tract. Since Caco-2 cells closely mimic the structure and function of mature intestinal epithelial cells, they are commonly used for such studies. In this research, human colorectal adenocarcinoma Caco-2 cells were employed as the model system. This choice was made based on the assumption that ingestion is the primary route of microplastic intake in humans. The Caco-2 monolayer is particularly useful for investigating the integrity and permeability of the intestinal barrier against microplastics. The objective of this study was to evaluate the toxicological effects of polyethylene terephthalate and polyethylene microplastics. Two different particle sizes, 1 µm and 2.6 µm, were used for the exposure. Cells were exposed for 72 h, and the toxicological effects were assessed through cell viability, the generation of reactive oxygen species, apoptosis, and autophagy.

## 2. Materials and Methods

### 2.1. Cell Culture

Caco-2 cell lines were sourced from ATCC (Manassas, VA, USA) and cultured in RPMI medium enriched with 10% fetal bovine serum, 100 µg/mL streptomycin, 100 U/mL penicillin, and 1% L-glutamine. The cells were kept at 37 °C in a humidified atmosphere containing 5% CO_2_, with subculturing carried out every 3–4 days. To assess the cytotoxicity of MPs, Caco-2 cells were rinsed with PBS, detached using trypsin, and counted. Afterward, they were plated in 96-well plates at a concentration of 1.5 × 10^4^ cells per well and left to attach for 24 h before their exposure to MPs.

### 2.2. Exposure Conditions

MP solutions were prepared following the methods outlined by [23,24]. The stock solution of MPs was autoclaved for 40 min before being added to the cell culture. PE (1 µm) and PET (1 µm and 2.6 µm) were then incubated with the cells for 72 h.

### 2.3. CCK8 Assay

Cell viability was evaluated using the CCK-8 colorimetric assay (Dojindo Molecular Technologies, Rockville, MD, USA, CK04). A total of 1500 cells were seeded in 96-well plates, followed by the addition of CCK-8 reagents. After 72 h of incubation, absorbance was measured at 450 nm using a Varioskan Flash microplate reader (Thermo Scientific, Waltham, MA, USA). To ensure the accuracy of the results, the experiments were conducted with eight independent replicates.

### 2.4. ROS Assay

Intracellular ROS levels were evaluated using the 2,7dichlorofluorescin diacetate (DCFH-DA) assay ( Cat# 35845-1G, Sigma Aldrich, St. Louis, MO, USA).

### 2.5. Annexin-V Assay

Apoptotic levels in Caco-2 cells were determined using an Annexin V kit conjugated with fluorescein (Millipore, Burlington, MA, USA). Following annexin V staining, the proportion of apoptotic cells was analyzed using a Guava easyCyte cytometer, following the manufacturer’s guidelines. Briefly, Caco-2 cells were plated in 6-well plates at a density of 2 × 10^5^ cells per well and exposed to different experimental conditions. After 72 h, cells from each experimental group were collected from culture plates and stained using an Annexin V-FITC solution along with 7-AAD before cytometric analysis was performed.

The kit utilizes Annexin V and 7-AAD dyes to distinguish a broad range of cells undergoing apoptosis from those that are not. Annexin V, labeled in green, specifically binds to phosphatidylserine on the external membrane of apoptotic cells. Meanwhile, 7-AAD, shown in red, penetrates cell membranes and stains the DNA of cells in the late apoptotic and dead stages. Through this staining process, it becomes possible to categorize cells into four populations: early apoptotic cells, non-apoptotic cells, necrotic cells, and late apoptotic or dead cells. In our experiment, late and early apoptotic cells were combined [25].

### 2.6. Protein Extraction

After removing the culture medium, adherent Caco-2 cells were rinsed with 1X PBS (EuroClone, S.p.A., Pero, Italy). The cells were then lysed using a specific buffer containing Nonidet-P40 (NP-40), sodium deoxycholate in PBS, and sodium dodecyl sulfate, supplemented with protease inhibitors, including sodium orthovanadate (Na_3_VO_4_), sodium fluoride (NaF), phenylmethylsulfonyl fluoride (PMSF), and a Roche protease inhibitor cocktail. Lysis was performed by scraping and carefully resuspending the cells, followed by centrifugation at maximum speed of 16.400 rpm for 5 min in a refrigerated centrifuge (Eppendorf 5417R, Milan, Italy) at 4 °C. The protein concentration in the supernatant was quantified using the Bradford assay. Unless stated otherwise, all reagents were sourced from Sigma-Aldrich (St. Louis, MO, USA) [26].

### 2.7. Western Blotting (WB)

As described by [27], protein lysates (10–40 µg per sample) were separated using 9% and 15% SDS-polyacrylamide gels. The separated proteins were subsequently transferred onto polyvinylidene difluoride (PVDF) membranes (Amersham Pharmacia Biotech, Buckinghamshire, UK) under a constant current of 280 mA for 3 h at 4 °C. To prevent non-specific binding, the membranes were blocked for 3 h under agitation in a solution containing 5% milk in Tris-buffered saline (TBS) with 150 mM NaCl, 10 mM Tris–HCl (pH 7.6), and 0.25% Tween-20 (Sigma-Aldrich). Primary antibody incubation (Supplementary Table S1) was carried out overnight at 4 °C with gentle agitation. After successive washes with T-TBS (TBS containing 0.25% Tween-20) followed by TBS alone, the membranes were incubated with the corresponding secondary antibodies (Supplementary Table S1). The filters were then washed three times in T-TBS, and immunocomplexes were visualized using the enhanced chemiluminescence (ECL) detection system for Western blotting (Amersham Pharmacia Biotech). Statistical analyses were conducted based on three independent Western blot experiments per sample, and band intensity quantification was performed using ImageJ version 1.53 K software.

### 2.8. Immunofluorescence (IF) Analysis

Following exposure to MPs, Caco-2 cells were cultured in 24-well plates. In brief, the cells were fixed at room temperature (20–23 °C) for 15 min using a 4% formaldehyde solution (Sigma-Aldrich). Permeabilization was then carried out with 0.3% Triton X-100 (Roche, Basel, Switzerland) for 5 min. To reduce non-specific binding, a blocking solution consisting of 5% FBS in PBS with 0.1% Triton X-100 was incubated for 1 h at room temperature [24]. Cells were subsequently incubated overnight at 4 °C with primary antibodies (Supplementary Table S1). After three PBS washes, the appropriate secondary antibody, diluted in the blocking solution, was added for 1 h at room temperature. For nuclear staining, DAPI mounting medium (ab104139, ABCAM, Cambridge, UK) was used, and fluorescence images were captured with a Leica fluorescence microscope (Wetzlar, Germany).

### 2.9. Statistical Analysis

Statistical analysis was performed using a one-way ANOVA, followed by Tukey’s post hoc test. Data were analyzed with GraphPad Prism version 5.01 (GraphPad, San Diego, CA, USA). A *p*-value of less than 0.05 was regarded as statistically significant. The results are expressed as the mean ± standard error of the mean (SEM).

## 3. Results

### 3.1. Microplastics Reduce the Proliferative Capacity of Caco-2 Cells

**Figure 1:**
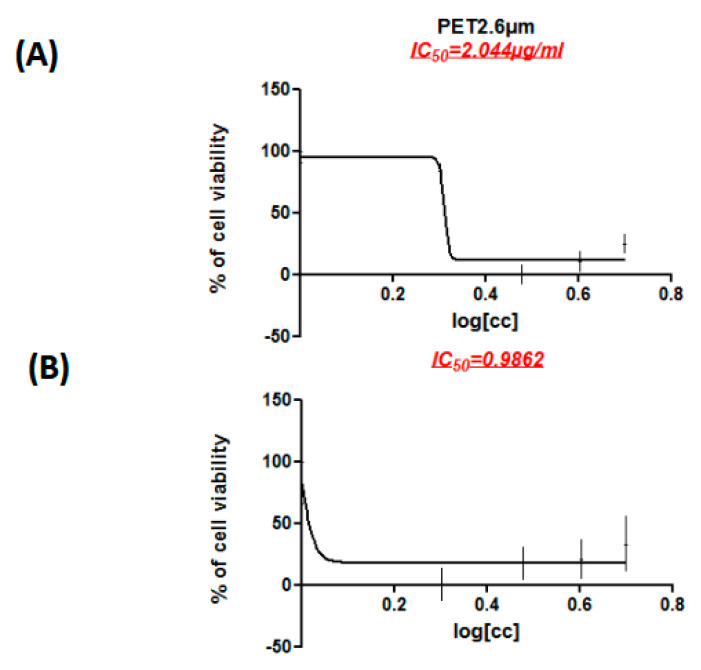
MPs (PET 2.6 µm and PE 2.6 µm) exposure decrease cell viability of Caco-2 cell line. (**A**): Standard curves and IC50 values of PET 2.6µm for Caco-2 cell line. (**B**) Standard curves and IC50 values of PE 2.6 µm for Caco-2 cell line. Calculation of IC50 values in Graph Pad Prism. Fraction of alive cells [%] is provided on the vertical axis and the log (concentration) on the horizontal axis. The IC50 is the concentration at which the curve passes through the 50% inhibition level. CCK8 assay was performed to measure cell viability.

Caco-2 cell cultures were treated with two types of MPs: PET with particle sizes smaller than 1 µm and 2.6 µm and PE with particle sizes below 2.6 µm. The exposure duration spanned from 24 to 72 h, with the MPs’ concentrations varying between 10 µg/mL and 40 µg/mL. The data obtained demonstrated concentration-dependent effects, with minimum active concentration (MAC) values of 2.04 µg/mL for PET (2.6 µm) (Figure 1A) and 0.98 µg/mL for PE (Figure 1B). A further analysis of cell viability using the CCK-8 assay revealed a significant reduction in the proliferation of Caco-2 cells following exposure to PET 1, PET 2.6µm, and PE. These results highlight the significant effect of microplastics (MPs) in reducing the proliferative capacity of Caco-2 cells.

### 3.2. MPs Induce Increased Oxidative Stress, Leading to Enhanced ROS Production

**Figure 2:**
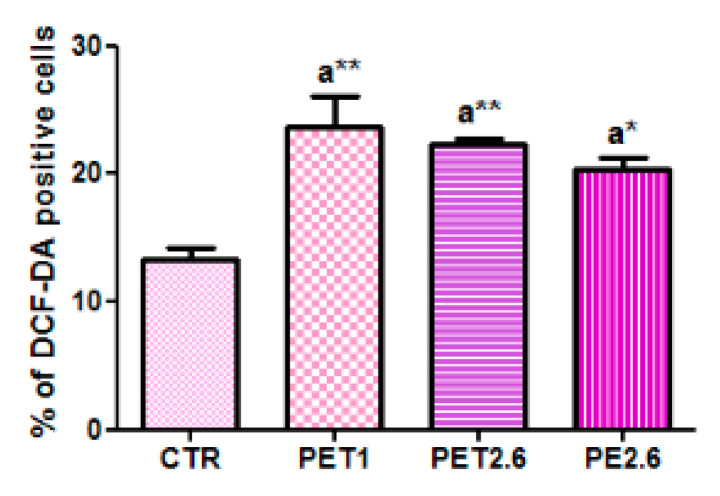
ROS generation in Caco-2 cells exposed for 72 h to MPs (PET 1 µm, PET 2.6 µm and PE 2.6 µm) at IC50 concentrations. Histogram showing the percentage of DCF-DA positive cells. All values are expressed as means ± standard deviation (* *p* < 0.05, ** *p* < 0.01). Statistical significance was evaluated by ANOVA (at least *p* < 0.05) followed by Tukey test for multigroup comparison. All the experiments were performed in triplicate.

The accumulation of ROS in Caco-2 cells exposed to MPs was evaluated using the DCFH-DA dye. As shown in Figure 2, after 72 h of MP exposure, the fluorescence levels of DCFH-DA significantly increased in treated CaCo2 cells (*p* < 0.05, *p* < 0.01), as measured by the Guava^®^ easyCyte™ flow cytometer (Luminex Corporation, Austin, TX, USA). These results indicate that MPs trigger excessive ROS production.

### 3.3. Microplastic-Induced Apoptotic Effects in Caco-2 Cells

**Figure 3:**
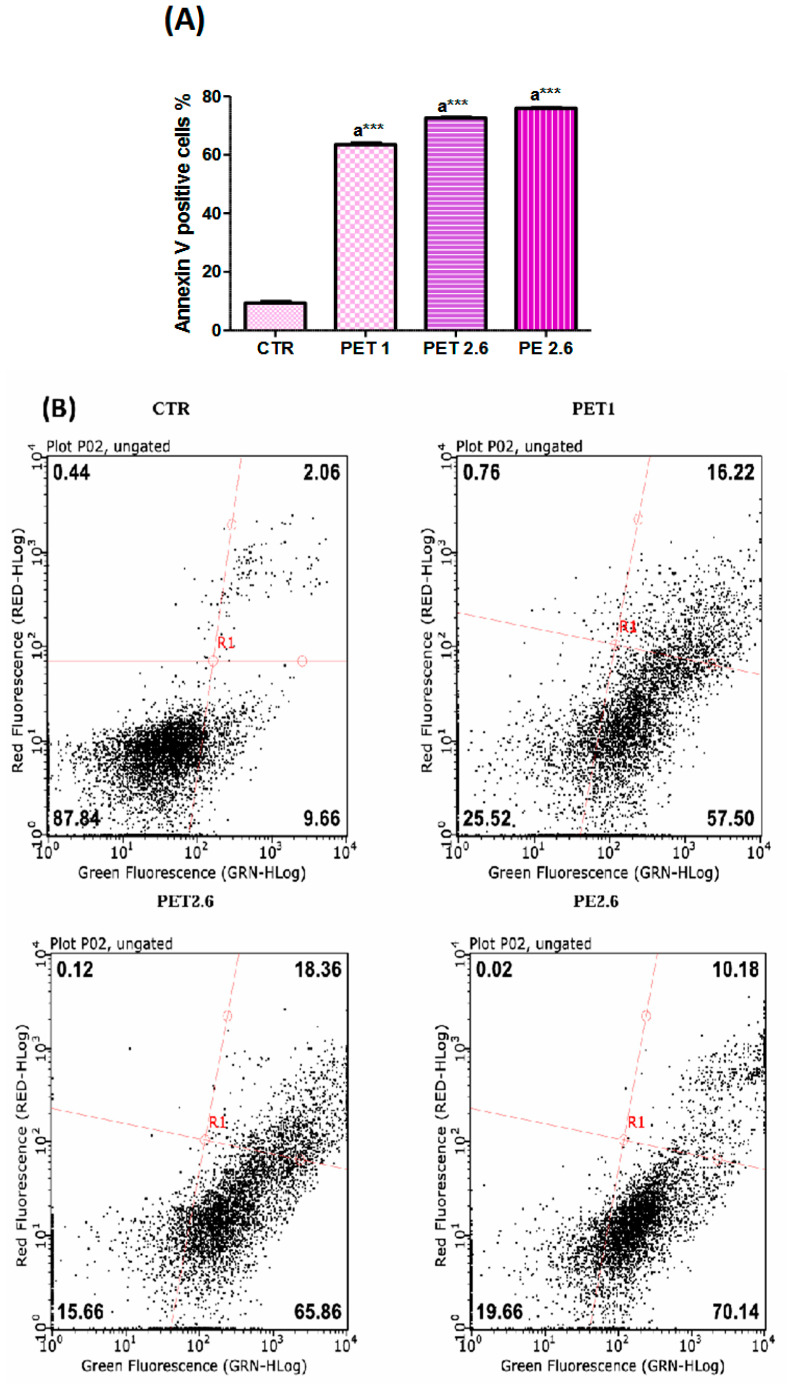

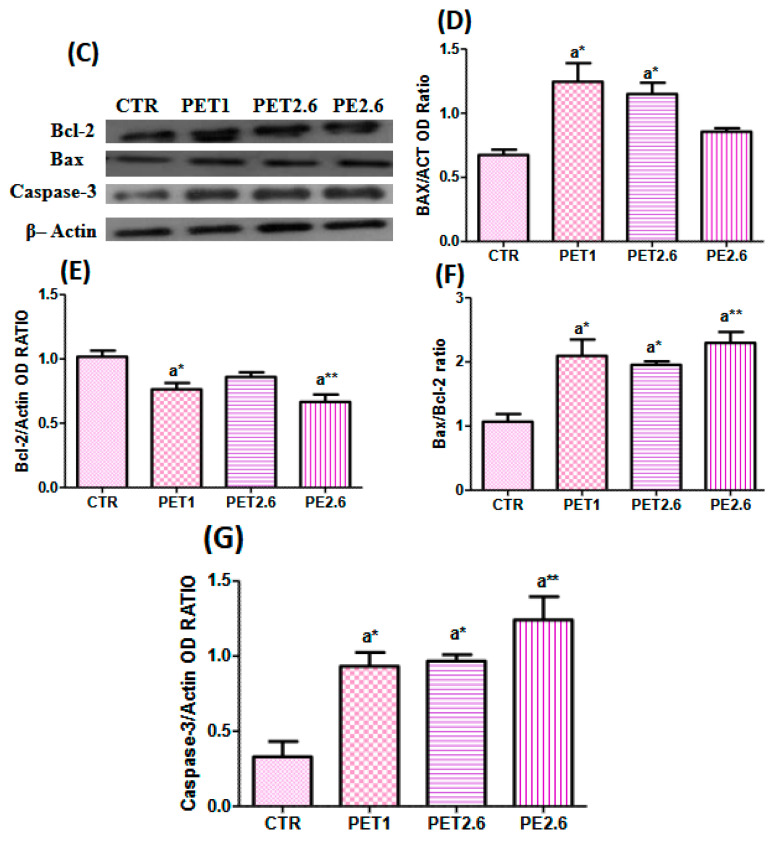
Analysis of apoptosis in controls, PET 1µm, PET 2.6 µm and PE 2.6 µm. Caco-2 cells were treated with each MP at its IC50 concentration for 72 hours. (**A**) The histogram shows the global percentage of Annexin V-positive cells. (**B**) Representative apoptosis FACS analysis. The experiments were carried out after 72h of treatment with MPs. The assay allows the identification of early (Annexin V + and 7ADD − and late apoptosis (Annexin V + and 7ADD +). (**C**) WB analysis showing the expression of Caspase-3 (35kDa), Bax (23 kDa) and Bcl-2 (26 kDa) in Caco-2 exposed to MPs. (**D**) Histogram showing the relative level of Bax associated with apoptosis. (**E**) Histogram shows the relative level of Bcl-2. (**F**) Histogram shows the Bax/Bcl-2 ratio. Data were normalized with beta-Actin (44kDa) and reported as Bax/Bcl-2 ratio. (**G**) Histogram showing the relative level of Caspase-3. Proteins levels were quantifed using ImageJ program. Data were normalized with beta-Actin and reported as OD ratio. All the values are expressed as means ± SEM. Asterisks indicate a signifcant difference from the respective control (*: *p* < 0.05), (** *p* < 0.01) and (***: *p* < 0.001) after one-way ANOVA using Tukey’s Post Hoc test.

Apoptotic rates were analyzed using flow cytometry (Figure 3B). The results demonstrated that microplastics increased apoptosis in all the analyzed conditions (Figure 3A). Specifically, the apoptotic cell populations were significantly higher in the MP-treated groups compared to the control, suggesting that microplastic exposure may promote cell death through apoptotic mechanisms. This trend was observed across all experimental conditions, further supporting the hypothesis that MPs induce cellular stress and contribute to apoptosis.

Bcl-2, Bax, and Caspase-3 were chosen as key markers for apoptosis (Figure 3C). The Western blot analysis (Figure 3D) revealed that the protein levels of Bax were elevated in the Caco-2 cells treated with PET1 and PET2.6 (*p* < 0.05). In contrast, Bcl-2 levels (Figure 3E) decreased in the cells treated with PET1 (*p* < 0.05) and PE (*p* < 0.01). As a result, the Bax/Bcl-2 ratio significantly increased across all conditions (Figure 3F). Additionally, caspase-3 levels were elevated in all the conditions tested (Figure 3G), with PET showing a significant increase (*p* < 0.05) and PE showing a more pronounced increase (*p* < 0.01).

### 3.4. Impact of PET and PE on the Autophagic Process

**Figure 4:**
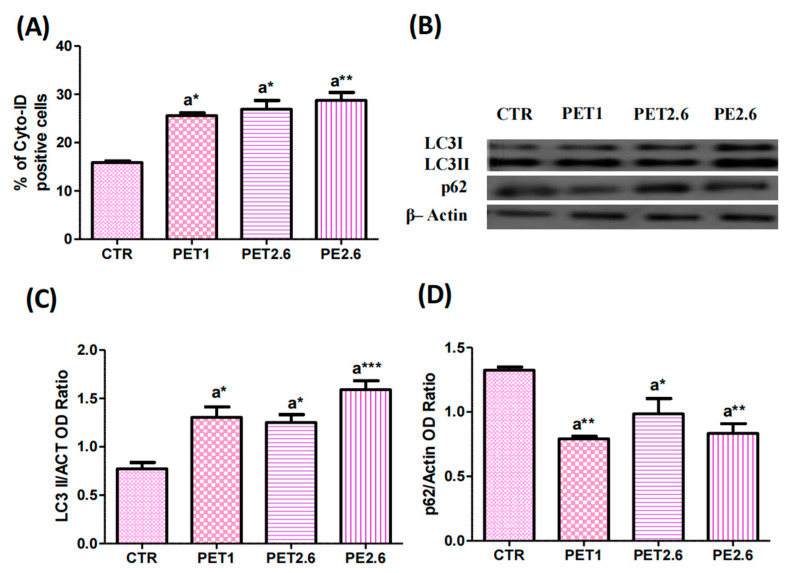
Assessment of autophagic flux in Caco-2 cells exposed to microplastics (PET 1 µm, PET 2.6 µm, and PE 2.6 µm) using Cyto-ID assay and Western blot analysis. (**A**) Histogram showing the percentage of Cyto-ID positive cells. (**B**) WB analysis shows the expression of LC3-I (16 kDa), LC3-II (14kDa) and p62 (62 kDa) in Caco-2 exposed to MPs. (**C**) The histogram shows the LC3-II level. Data were normalized with beta-Actin (44 kDa). (**D**) Histogram showing the relative level of p62. Proteins levels were quantified using the ImageJ program. Data were normalized with beta- Actin and reported as OD ratio. All the values are expressed as means ± SEM. Asterisks indicate a significant difference from the respective control (*: *p* < 0.05), (** *p* < 0.01) and (***: *p* < 0.001) after one-way ANOVA using Tukey’s Post Hoc test.

**Figure 5:**
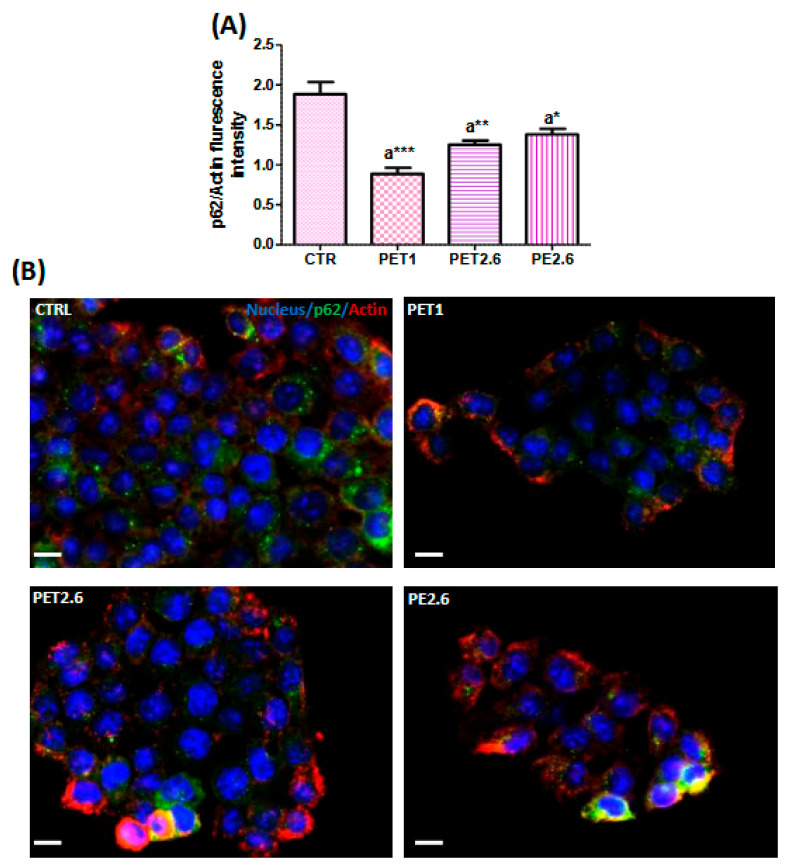

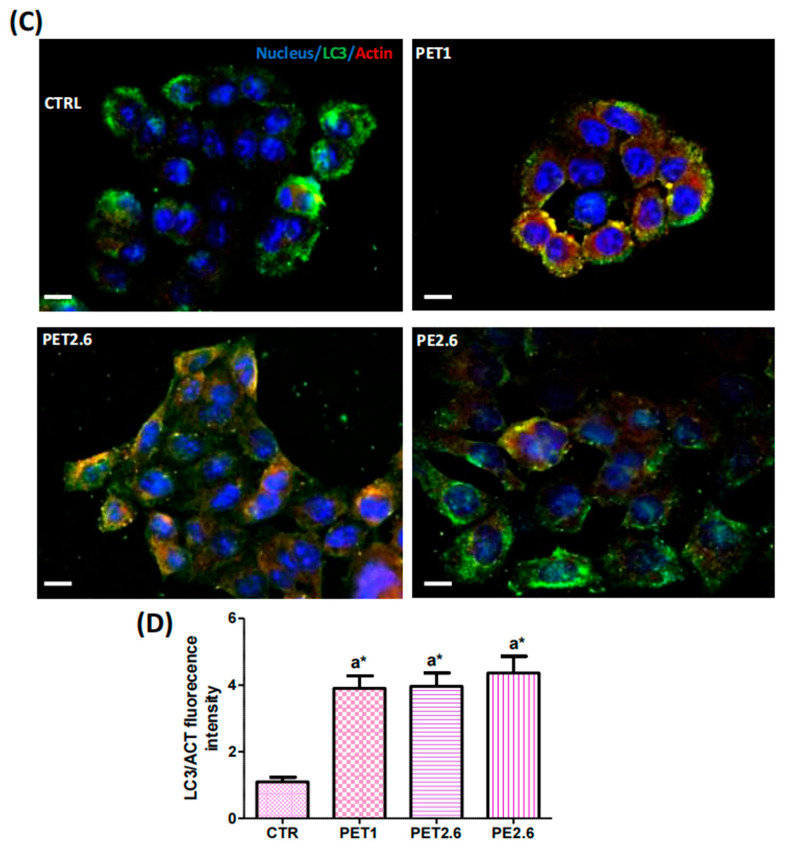
Autophagic flux analysis by immunofluorescence. (**A**) the histogram shows the quantification of p62 fluorescence signal intensity. (**B**) p62 (green), Actin (Red) immunolocalization Caco-2 cells treated with MPs. Cell nuclei were stained with DAPI (blue). The images were captured at ×20 magnification. Scale bars represent 20 µm. Data were normalized with the signal of Actin using ImageJ. All values are expressed as means ± standard deviation. PET 1, PET 2.6 and PE 2.6 Vs CTRL: (*: *p* < 0.05, ** *p* < 0.01 and ***: *p* < 0.001). (**C**) Representative micrographs of LC3 immunostaining (green), and Actin (red) on Caco-2 cultures treated with MPs. Cell nuclei were stained with DAPI (blue). (**D**) The graph shows the quantification of LC3 fluorescence signal intensity Using Tukey’s Post Hoc test. Asterisks indicate a significant difference from the respective control (*: *p* < 0.05 ) after one-way ANOVA using Tukey’s Post Hoc test.

**Figure 6:**
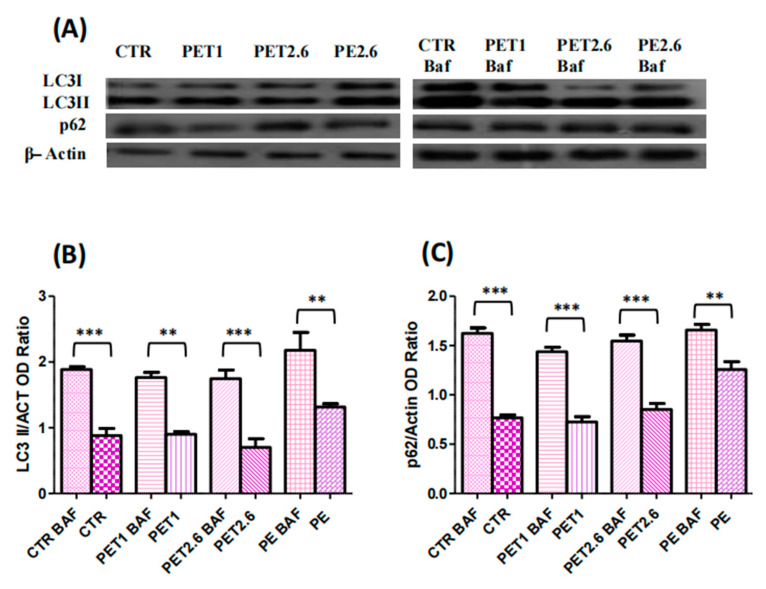
Co-treatment with bafilomycin A1. Caco-2 cells were exposed to MPs at their IC50 concentrations for 72 hours in the presence or absence of bafilomycin A1. (**A**) WB analysis showing the expression of LC3-I (16 kDa), LC3-II (14 kDa) and p62 (62 kDa) in Caco- 2 exposed to MPs in the presence and absence of Bafilomycin A1. (**B**) Histogram shows the LC3-II level. Data were normalized with beta-Actin (44 kDa). (**C**) Histogram showing the relative level of p62. Protein levels were quantified using the ImageJ program. Data were normalized with beta-Actin and reported as OD ratio. All the values are expressed as means ± SEM. Asterisks indicate a signifcant difference from the respective control (** *p* < 0.01) and (***: *p* < 0.001) after one-way ANOVA using Tukey’s Post Hoc test.

To investigate the potential role of autophagy in cellular responses to microplastics (MPs), our study focused on understanding how MPs affect cellular processes through autophagic mechanisms. We used cytochalasin D to disrupt the actin cytoskeleton and assessed autophagic activity using complementary methods. A Western blot analysis of autophagy-related proteins LC3 and p62 was performed under conditions with and without bafilomycin A1 (Baf A1), a selective inhibitor of vacuolar-type H^+^-ATPase that blocks the fusion of autophagosomes with lysosomes. Compared to the control group, LC3-II levels were significantly elevated in Caco-2 cells treated with PET 1µm, PET 2.6µm (*p* < 0.05), and PE (*p* < 0.001), while p62 levels were significantly reduced in these same groups (PET 1µm: *p* < 0.01, PET2.6µm: *p* < 0.05, PE: *p* < 0.01), indicating enhanced autophagic flux (Figure 4B–D). These findings were supported by the Cyto-ID^®^ Autophagy Detection assay, which uses a cationic amphiphilic tracer dye to label autophagic vacuoles. As shown in Figure 4A, the Caco-2 cells treated with PET1, PET2.6 (*p* < 0.05), and PE (*p* < 0.01) exhibited a significant increase in autophagic vacuoles compared to the control group. We further confirmed these results through immunofluorescence imaging, which revealed distinct localization patterns of LC3 and p62. A quantitative analysis showed a significant increase in LC3-II fluorescence intensity in PET- and PE-treated cells (*p* < 0.05), while p62 fluorescence decreased across all conditions (Figure 5A–D), reinforcing the conclusion of increased autophagic activity. To validate the presence of active autophagic flux, we evaluated LC3-II and p62 expression in the presence of Baf A1. As expected, the Baf A1 treatment led to a marked accumulation of both LC3-II and p62 (Figure 6A–C), consistent with the blockade of autophagosome degradation. The increased LC3-II reflects autophagosome accumulation, while elevated p62 suggests impaired degradation, confirming that MPs stimulate autophagy and alter degradation pathways in Caco-2 cells.

## 4. Discussion

Microplastics are widespread in marine, freshwater, terrestrial, and atmospheric environments, raising concerns about their potential risks to human health through ingestion and inhalation. In particular, their ability to adsorb organic pollutants may amplify their toxic effects within the body. Since the gastrointestinal system is a primary site of exposure to microplastics, it is crucial to understand how digestive processes influence their potential toxicity to the intestines, an area that remains poorly explored. Caco-2 cells were chosen for this study due to their relevance in modeling intestinal epithelium interactions. Previous research has shown that microplastics can directly engage with intestinal cells, while nanoscale plastics have the capability to pass through the digestive system and potentially be absorbed [28,29,30]. In this regard, Caco-2 cells were utilized to assess the potential harmful impacts of PET and PE, specifically in terms of cell viability and oxidative stress, providing a relevant model to investigate microplastic-induced toxicity in the intestinal epithelium. Indeed, after 72 h of MP exposure, the results indicated that Caco-2 cells displayed increased susceptibility to PE, as evidenced by its lower minimum active concentration (MAC) of 0.98 µg/mL, highlighting a stronger cellular reaction to this microplastic compared to PET. The CCK-8 assay indicated a considerable impact on cell proliferation due to exposure. Notably, after 72 h of treatment, both types of MPs induced a significant reduction in the proliferation rate, highlighting a distinct cellular reaction. Our results align with the study by [31], which demonstrated that the exposure of Caco-2 cells to PET decreases cell viability, as well as with the study by [32], which also showed that PE reduces cell viability after 24 h of exposure. Moreover, ref. [33] reported a 20–30% decline in cell viability after 48 h of exposure to polyethylene. In contrast, ref. [34] found no significant effects when using an inverted cell culture model with Caco-2 cells.

To identify the factors responsible for this increase, we measured the intracellular ROS levels using a DCF-DA assay. In fact, several studies across different polymer types and cell lines have reported an increase in reactive oxygen species (ROS) production after exposure to microplastics [31,32,33,35,36]. Consistent with these findings, our results also revealed an increase in ROS production in Caco-2 cells exposed to both PET and PE, further supporting the growing body of evidence linking microplastic exposure to oxidative stress. Building on the observed changes in cell viability and oxidative stress, we next investigated the potential induction of apoptosis in Caco-2 cells exposed to PET and PE. Apoptotic pathways are crucial for understanding the long-term effects of microplastic exposure, as they may contribute to cellular dysfunction. To explore this, we assessed apoptotic markers, including Annexin V staining and a Western blot analysis of key apoptosis-related proteins, such as Bcl-2 and Bax. Our study demonstrated that exposure to PET and PE microplastics not only affected cell viability and oxidative stress but also induced a significant increase in apoptosis, highlighting the toxic effects of these microplastics on intestinal epithelial cells. Our findings are consistent with those of [37], who reported that polystyrene triggers apoptosis in Caco-2 cells. Additionally, ref. [38] reported a slight increase in the Bax/Bcl-2 ratio in Caco-2 cells after polystyrene treatment. Furthermore, ref. [24] demonstrated that exposure to 2.6 µm PET microplastics led to increased apoptosis in adipose-derived mesenchymal stromal cells. Likewise, ref. [39] observed apoptotic effects in AGS cells, a model for human gastric adenocarcinoma. In addition, ref. [40] reported that polystyrene microplastics induced apoptosis in HepG2 cells. Moreover, the combined exposure of polystyrene nanoplastics induced apoptosis in Caco-2 cells through ER stress-mediated mitochondrial damage, oxidative stress, and the activation of the caspase family [41]. Additionally, it is important to consider the interaction between apoptosis and autophagy in response to microplastic exposure. Autophagy, a process that maintains cellular homeostasis by degrading damaged organelles and proteins, can be either protective or promotive of cell death, depending on the context. Recent studies suggest that microplastics can modulate autophagic pathways, which may further exacerbate cellular damage and influence the apoptotic response in intestinal epithelial cells. In this context, we assessed the autophagic process by measuring Cyto-ID levels and analyzing the protein expression of LC3-II and p62 through immunofluorescence and Western blotting. Our findings showed an increase in Cyto-ID, suggesting a heightened autophagic flux. Additionally, our results revealed an increase in LC3-II levels and a reduction in p62, confirmed through both Western blot and immunofluorescence assays. These findings collectively support the activation of autophagy. Our findings align with those of [38], who showed that the treatment of Caco-2 cells with polystyrene, accompanied by an increase in LC3-II, indicates that polystyrene exposure may trigger autophagic cell death in Caco-2 cells. Similarly, it has been shown that polystyrene induced autophagic cell death in both bronchial epithelial cells and human umbilical vein endothelial cells [42,43]. Increased levels of autophagy can reflect either a boost in the autophagic process itself or a disruption in the lysosomal processing that occurs after autophagosome formation, or both factors in tandem. To investigate this, we employed Baf A1, a specific inhibitor of vacuolar-type H^+^-ATPase, which blocks the fusion of autophagosomes and lysosomes, thereby hindering autophagy at a later stage. We evaluated the expression of LC3-II isoforms and p62 in conditions with and without Baf A1. Our results showed an increase in both LC3 and p62 in the presence of Baf A1, indicating active autophagic flux. The rise in LC3 levels suggests enhanced autophagosome formation, indicating that MPs stimulate autophagy initiation. Meanwhile, the accumulation of p62 points to a blockage in the autophagic flux. Although autophagosome formation was induced, the degradation of cargo (including p62) within autolysosomes was impaired, likely due to the presence of MPs. These findings underscore the intricate effects of MPs on autophagy and highlight the need to consider both the initiation and flux stages when examining the modulation of autophagy by MPs.

## 5. Conclusions

This study offers important perspectives on the toxicological effects of microplastic exposure on intestinal epithelial cells. Our findings demonstrate that both PET and PE microplastics significantly impair Caco-2 cell viability by inducing oxidative stress, apoptosis, and autophagy. The observed increase in ROS levels and apoptosis-related protein expression suggests that MPs disrupt cellular homeostasis, potentially leading to intestinal dysfunction. Furthermore, the activation of autophagic flux indicates a cellular attempt to counteract MP-induced stress, but it may also contribute to cell death under prolonged exposure. Given the increasing presence of MPs in food and water sources, these results underscore the importance of regulatory strategies aimed at minimizing microplastic exposure. Policymakers should consider these findings when establishing guidelines to limit the contamination of food and water with microplastics. To strengthen the understanding of the long-term health risks associated with MPs, additional studies are highly recommended. These should explore different exposure durations and include a broader range of cell types such as HT29 cells, primary human intestinal cells, and immune cells like macrophages. The use of animal models such as mice and rats could also provide more comprehensive insights into the systemic effects of microplastics. Expanding research to include a diversity of models would allow for a more thorough assessment of microplastic toxicity, ultimately supporting the development of informed public health policies and regulations.

## Data Availability

The data presented in this study are available on request from the corresponding author. The data are not publicly available due to privacy restrictions.

## Supplementary Materials

The following supporting information can be downloaded at https://www.mdpi.com/article/10.3390/ijerph22060922/s1, Supplementary Table S1: List of all the used antibodies.
