# Supplementary material for: Impact of Environmental Microplastic Exposure on Caco-2 Cells: Unraveling Proliferation, Apoptosis, and Autophagy Activation
Source: Int J Environ Res Public Health. 2025 Jun 11;22(6):922. doi: 10.3390/ijerph22060922 (PMC12193311; doi:10.3390/ijerph22060922)
Supplement: Supplementary file 1 [file ijerph-22-00922-s001.zip › ijerph-3650630-supplementary.pdf]

Impact of Environmental Microplastic Exposure on Caco-2 Cells: Unraveling Proliferation, Apoptosis, and Autophagy Activation

Hana Najahi [1,2,*], Nicola Alessio [3], Massimo Venditti [3], Gea Oliveri Conti [4], Margherita Ferrante [4], Giovanni Di Bernardo [3], Umberto Galderisi [3], Sergio Minucci [3] and Mohamed Banni [1,2]

[1] Laboratory of Agrobiodiversity and Ecotoxicology LR21AGR02, Sousse University, Chott-Mariem, Sousse 4042, Tunisia
[2] Higher Institute of Biotechnology, Monastir University, Monastir 1002, Tunisia
[3] Department of Experimental Medicine, "Luigi Vanvitelli" Campania University, 81038 Napoli, Italy
[4] Environmental and Food Hygiene Laboratory (LIAA), Department of Medical, Surgical Sciences and Advanced Technologies G. F. Ingrassia, Catania University, Via Santa Sofia 87, 95123 Catania, Italy
* Correspondence: hana.najahi@yahoo.fr

Supplementary Table S1: List of all the used antibodies.

Antibody	WB Dilution	IF Dilution	Source
Beta-Actine	1:10000	1 :100	Elabscience Biotechnology, Wuhan, China #E-AB-20031
LC-3	1:500	1:100	Sigma-Aldrich,Milan,Italy #L7543
p62	1:500	1:100	Elabscience Biotechnology, Wuhan, China #E-AB-63539
Goat anti-mouse HRP	1:5000	-	Sigma aldrich,Milan, Italy #AP130P
Goat anti-rabbit HRP	1:5000	-	Sigma-Aldrich, Milan, Italy #AP307P
Goat anti-rabbit	-	1:500	Thermo Fisher Scientific, Waltham, Ma, USA #A32731
Alexa Fluor 647 goat anti-mouse		1 :500	Thermo Fisher Scientific, Waltham, Ma, USA #A21236
Bcl-2	1:1000	1:100	Elabscience Biotechnology, Wuhan, China #E-AB-60012
Bax	1:1000	1:100	Elabscience Biotechnology, Wuhan, China #E-AB-13814
Active Caspase-3	1:1000	1:100	Elabscience Biotechnology, Wuhan, China #E-AB-22115
